# Biochemical Screening of Intellectually Disabled Patients: A Stepping Stone to Initiate a Newborn Screening Program in Pakistan

**DOI:** 10.3389/fneur.2019.00762

**Published:** 2019-07-17

**Authors:** Muhammad Wasim, Haq Nawaz Khan, Hina Ayesha, Susanna M. I. Goorden, Frederic M. Vaz, Clara D. M. van Karnebeek, Fazli Rabbi Awan

**Affiliations:** ^1^Health Biotechnology Division, National Institute for Biotechnology and Genetic Engineering (NIBGE), Faisalabad, Pakistan; ^2^Pakistan Institute of Engineering and Applied Sciences, Islamabad, Pakistan; ^3^Department of Pediatrics, DHQ and Allied Hospitals, Faisalabad Medical University (FMU/PMC), Faisalabad, Pakistan; ^4^Laboratory Genetic Metabolic Diseases, Department of Clinical Chemistry, Amsterdam University Medical Centers, University of Amsterdam, Amsterdam, Netherlands; ^5^Departments of Pediatrics and Clinical Genetics, Emma Children's Hospital, Amsterdam University Medical Centers, University of Amsterdam, Amsterdam, Netherlands

**Keywords:** inborn errors of metabolism (IEMs), intellectual disability, newborn screening (NBS), evidence based medicine (EBM), homocystinuria, Pakistan

## Abstract

Inborn errors of metabolism (IEMs) are rare group of genetic disorders comprising of more than 1,000 different types. Around 200 of IEMs are potentially treatable through diet, pharmacological and other therapies, if diagnosed earlier in life. IEMs can be diagnosed early through newborn screening (NBS) programs, which are in place in most of the developed countries. However, establishing a NBS in a developing country is a challenging task due to scarcity of disease related data, large population size, poor economy, and burden of other common disorders. Since, not enough data is available for the prevalence of IEMs in Pakistan; therefore, in this study, we set out to find the prevalence of various treatable IEMs in a cohort of intellectually disabled patients suspected for IEMs, which will help us to initiate a NBS program for the most frequent IEMs in Pakistan. Therefore, a total of 429 intellectually disabled (IQ <70) patient samples were collected from Pakistan. A subset of 113 patient samples was selected based on the clinical information for the detailed biochemical screening. Advance analytical techniques like, Amino Acid Analyzer, GC-MS, UHPLC-MS, and MS/MS were used to screen for different treatable IEMs like aminoacidopathies, fatty acid β-oxidation disorders and mucopolysaccharidoses (MPS) etc. A total of 14 patients were diagnosed with an IEM i.e., 9 with homocystinuria, 2 with MPS, 2 with Guanidinoacetate methyltransferase (GAMT) deficiency and 1 with sitosterolemia. These IEMs are found frequent in the collected patient samples from Pakistan. Thus, present study can help to take an initiative step to start a NBS program in Pakistan, especially for the homocystinuria having highest incidence among aminoacidopathies in the studied patients, and which is amenable to treatment. This endeavor will pave the way for a healthier life of affected patients and will lessen the burden on their families and society.

## Introduction

Inborn errors of metabolism (IEMs) are phenotypically and genetically heterogeneous disorders which are caused by the deficiency of a protein (most often an enzyme or a transporter) that results in the accumulation of intermediary metabolites which cannot be processed further. Although IEMs are rare when taken individually, but collectively they form a significant group of disorders, which lead to morbidity and mortality of the affected patients (1–3). Overall, prevalence of IEMs is estimated to be 1 in 500 newborns (4). Up till now more than 1,000 IEMs have been reported and about 200 of such disorders are potentially treatable when diagnosed before the appearance of clinical symptoms (5–7). Affected individuals usually appear normal at the time of birth, but symptoms (e.g., poor feeding, seizures, lethargy etc.) may develop within hours to weeks depending upon the disease severity. However, if diagnosed earlier, it allows treatment at the pre-symptomatic stage, thus preventing the subsequent development of intellectual disability. Diagnosis of these disorders can be performed through newborn screening (NBS) programs, which have been established in many developed countries (7–9).

In Pakistan, the prevalence of IEMs is expected to be high due to the high rate of consanguinity (~70% in Pakistan), which plays a significant role in the inherited disorders due to the autosomal recessive inheritance pattern (10). In Pakistan, the common practice of marriages takes place within the closely related communities, tribes, castes and in the same ethnic groups. Thus, the risk of any inherited disease in the offspring of closely related parents is reasonably high. Moreover, research on IEMs is neglected in the developing countries like Pakistan due to the burden of infectious and other common metabolic disorders.

Most of the developed countries have NBS programs in place which can screen for different inherited disorders at the time of birth or soon after birth (11–16). However, sadly, there is still no local or national level NBS program for any of the IEM in Pakistan—a country with population of more than 220 million. Indeed, there are many challenges to setup a NBS program for IEMs in the developing countries (17, 18). There are several pros and cons to initiate a NBS program in the developing countries as summarized in Table 1.

**Table 1:** Pros and Cons to initiate a NBS program in the developing countries.

**Pros and Cons to establish a NBS program in the developing countries**	
**Pros**	**Cons**
•Fast screening of different metabolic disorders	•Requirement of technical staff and advance equipments
•Early diagnosis and treatment	•Every newborn baby should be screened
•Affected patients can live a healthy life	•Which disorders should be a part of NBS and which are not
•Minimize the burden of rare metabolic disorders from the society	•Should know about the incidence of specific disorders according to population
•Cost effective for the population screening	•Huge funding required to initiate a NBS program
•Small quantity of biofluids required for the screening of many disorders	•Specialist required in the field of pediatrics, metabolomics, clinical and biochemical genetics
•Pre-symptomatic diagnosis	•Collaboration with public and private hospitals
•Family planning and prenatal diagnosis	•False positive and unclear results
•Genetic counseling for the carriers of a specific disease	•Delayed diagnosis in case of false negative results

Recently, in the Hong Kong, it has been advocated that NBS program is essential for the screening of newborns for IEMs, thus setting a reference for policymakers to establish a government funded NBS program to reduce the burden of these disorders (19). Likewise, several studies in China showed substantial occurrence of IEMs with an estimated prevalence of 1 in 3,795 newborns (20). Recently, 364,545 newborns were screened for IEMs in Quanzhou region of China, which showed 130 different IEMs with an incidence of 1 in 2,804 (21). For detailed characterization of various IEMs at the biochemical and genetic levels, tandem mass spectrometry and next generation sequencing was effectively used in China (22, 23). Interestingly, Sri Lanka is the only developing country in Asia which provides free healthcare services through NBS to all its citizens (24). Recently, efforts for NBS for some of the IEMs have also been started in Bangladesh with the help of international collaborators (25). Conversely, India which has the second largest population in the world has the weakest NBS program which is still at an infancy stage (26). There are few reports from India on the screening of IEMs; which showed congenital hypothyroidism as the most frequent disorder (27–29). However, the accurate prevalence of different IEMs is still unknown in India owing to lack of national level policy and NBS program (30). Likewise, due to similar problems, Pakistan also lags behind in setting up an NBS program for any of the IEMs. Although we acknowledge the challenge that costs of treatment pose in a developing country such as Pakistan, many IEMs are treatable with nutritional therapy which is relatively affordable and accessible, and we do believe that this population also has a right to prevention through early diagnosis.

Therefore, in the current study, research was initiated on the identification and characterization of treatable IEMs with the help of international experts, clinicians, geneticists and biochemists, which will pave the way for establishing a NBS program in Pakistan.

Since, special diets, pharmacological, and other therapies are now available for several IEMs; these will be targeted in the identified patients. Thus, for the treatable IEMs, evidence based medicine (EBM) approach would be proposed. Many EBM approaches have been developed and survival rates of patients with such treatable IEMs have improved substantially in recent years. In 2012, 83 drugs were reported, which were used for the treatment of different IEMs (2, 31). Despite the rarity of these disorders, there is growing emphasis on the use of EBM approach in the treatment of such rare metabolic disorders.

Therefore, the aim of this study was to screen a cohort of intellectually disabled patients for the identification of treatable IEMs, and to determine their prevalence for generating data to initiate NBS program in Pakistan.

## Materials and Methods

This study was approved by the institutional ethical review committee (*National Institute for Biotechnology and Genetic Engineering, NIBGE, Faisalabad, Pakistan)*. Blood and urine samples were collected from the intellectually disabled patients (IQ <70), along with important clinical information from January 2016 to December 2018.

In the current study, sample size for the patients was calculated by an online tool “Sample Size Calculator” by creative research systems (www.surveysystem.com/sscalc.htm). By providing relevant information like; confidence interval (95%), confidence level (5%) and population size of infants from two cities (Lahore and Faisalabad, second and third largest cities) of Pakistan (7.6 million). The “Sample Size Calculator” tool showed that 384 patients were needed to conduct this study. Hence, for the effectiveness, we have enrolled the maximum number of patients possible in the given time period comprising of a total of 429 intellectually disabled patient samples (suspected for IEMs), including 5 ID patients from Swat, Pakistan.

For patient sample collection, Special Education Centers (SECs) were visited in the two major cities (Lahore and Faisalabad) of Punjab, Pakistan. Permission was taken from the local government and school management as well as informed written consent of parents was obtained prior to sample collection. In each SEC, biofluid (peripheral blood and urine) from each intellectually disabled patient was collected. A total of 18 public SECs have were visited; 11 in Faisalabad and 7 in Lahore. From these SECs and with the support of our clinical collaborator from government (DHQ/Allied) hospitals, Faisalabad, a total of 429 patient blood samples and 340 urine samples were collected. The ratio of boys to girls was 5:2. Plasma and serum were separated from the peripheral blood for the detailed biochemical analyses and saved at −20°C until further analysis. All the experimental analyses were mainly performed during 2018.

To ascertain the presence and estimate prevalence of treatable IEMs, biochemical analyses were started on the collected patient samples. All the patient samples (*n* = 429) were first analyzed through an analytical HPLC assay for the screening of aminoacidopathies at NIBGE, Faisalabad, Pakistan. From this cohort of all patients, 113 patient samples were selected on the basis of clinically important parameters like, consanguinity, ectopia lentis, hypopigmented hair, hyperactivity, aggressive behavior, developmental delayed milestones, and tall stature etc. For detailed biochemical screening analyses and advance analytical techniques (Amino Acid Analyzer (AAA), GC-MS, UHPLC-MS, and MS/MS) were used at the Amsterdam University Medical Centers (UAMC), University of Amsterdam, The Netherlands. For the diagnosis of various disorders, screening was started for different metabolites like; plasma amino acids, acylcarnitine, chenodeoxycholic acid, cholic acid, ursodeoxycholic acid, di- and tri-hydroxycholestanoic acid, guanidinoacetic acid, creatine, heparan sulfate, dermatan sulfate, keratan sulfate, cholesterol, cholestanol, campesterol, sitosterol, stigmasterol etc., to find the levels of these metabolites in the plasma samples of 113 intellectually disabled patients.

## Results

All the important clinical information of the selected 113 intellectually disabled patients is listed in Table 2. After the detailed biochemical analyses through advance techniques, four different IEMs were identified (homocystinuria, mucopolysaccharidoses (MPS), Guanidinoacetate methyltransferase (GAMT) deficiency and sitosterolemia) as listed in Table 3. Interestingly, 9 classical homocystinuria patients from 7 unrelated families were found with the high concentrations of plasma methionine (Mean = 888 μmol/L; Reference range: 11–43 μmol/L) and homocysteine (Mean = 290 μmol/L; Reference range: 6–19 μmol/L).

**Table 2:** Different clinically important parameters of intellectually disabled patients.

**Characteristics**	**No. (%)**
**Sex**	
Male	86 (76)
Female	27 (24)
**Age (years)**	
≤ 12(5-12)	54 (48)
>12 (12-23)	59 (52)
**Family history**	
Non-consanguinity	15 (13)
Consanguinity	98 (87)
**Phenotype**	
Intellectual disability	113 (100)
Mild (IQ = 55–69)	22 (19.4)
Moderate (IQ = 40–54)	62 (54.8)
Severe (IQ = 25–39)	23 (20.3)
Profound (IQ <25)	6 (5.3)
Delayed developmental milestones	113 (100)
Hyperactivity	36 (32)
Aggressive behavior	41 (36)
Epilepsy	8 (7)
Ataxia	9 (8)
Ectopia lentis	13 (11)
Tall stature	4 (3)
Hypopigmented hair	6 (5.3)
Pectus excavatum	3 (2.6)
Myopia	3 (2.6)

**Table 3:** Screening of different IEMs with advance analytical techniques and the prevalent disorders in the patient samples.

**Biochemical screening**	**Results with %**	**Techniques used**	**Disorders**
^*^Aminoacidopathies	9/429 (2.1%)^*^	HPLC, AAA, UHPLC-MS, MS/MS	Homocystinuria
Fatty acid β-oxidation disorders	0/113 (0%)	MS/MS	
Bile acids screening	0/113 (0%)	MS/MS	
Mucopolysaccharidoses	2/113 (1.76%)	MS/MS	Glycosaminoglycans (GAGs)
Purines and nucleotides	0/113 (0%)	MS/MS	
Sphingolipid	0/113 (0%)	MS/MS	
Sterols	1/113(0.88%)	GC, GC-MS	Sitosterolemia
Total homocysteine	0/113 (0%)	MS/MS	
Creatine metabolic disorders	2/113 (1.76%)	MS/MS	GAMT deficiency

* *Screening of aminoacidopathies was performed on all the 429 patient samples, so only in aminoacidopathies case the denominator was 429*.

*Remaining disorders were screened in the 113 selected patient samples, therefore for all the other groups of disorders the denominator was 113*.

There were 2 MPS patients with high concentrations of heparan sulfate (Mean = 316 μg/ml), keratan sulfate (Mean = 2,144 μg/ml) and dermatan sulfate (Mean = 85 μg/ml). The Whole Exome Sequencing (WES) of these MPS patients is in progress for the confirmation of disease at the genetic level. Moreover, 2 patients were also identified with GAMT deficiency with high concentration of guanidinoacetic acid (Mean = 15 μmol/L; Reference range: 0.4–1.8 μmol/L) and low concentration of creatine (Mean = 2.8 μmol/L; Reference range: 17–109 μmol/L), and 1 sitosterolemia patient was found with the high values of cholesterol (4,969 μmol/L; Reference range: 1,881–4,887 μmol/L), cholestanol (23 μmol/L; Reference range: 3.50–10 μmol/L), stigmasterol (17 μmol/L; Reference range: 0–10 μmol/L), campesterol (173 μmol/L; Reference range: 0–23 μmol/L) and sitosterol (325 μmol/L; Reference range: 0–16 μmol/L). Overall in this cohort of intellectually disabled patients, 14 patients were diagnosed with four distinct types of IEMs.

## Discussion

Overall the frequency of IEMs causing in the intellectual disability in different populations is 1 to 3% (32–34). Unfortunately in Pakistan very limited data is available for IEMs in general, let only frequency (35, 36), due to lack of any local, provincial or national level NBS program. For this reason, we initiated to screen intellectually disabled patients who were suspected for IEMs from the second (Lahore) and third (Faisalabad) largest cities of Pakistan. This comprehensive biochemical screening study of a subset of 113 intellectually disabled patients (taken from a bigger cohort of 429 intellectually disabled patients) will be a stepping stone to initiate a NBS program in Pakistan. Hence, screening was started for various treatable IEMs like aminoacidopathies, MPS, fatty acid β-oxidation disorders, creatine metabolic disorders etc. According to these screening analyses, prevalence of IEMs in the studied cohort from Pakistan appeared reasonably high (3.26%), which could be due to high rate of consanguinity.

In the identified IEMs in our cohort, homocystinuria was found as the most frequent aminoacidopathy so far in the intellectually disabled patient cohort from Punjab, Pakistan. Other studies have also reported this condition as the second most prevalent IEM in different populations after phenylketonuria (37–39). In our cohort we did not find any patient with phenylketonuria., but this could be due to the relatively limited sample size for a rare disease study. The reported incidence of homocystinuria varies from 1 in 344,000 worldwide to 1 in 65,000 in the Ireland, with highest in the Qatar as 1 in 1,800 (37, 40, 41). In the present study, 9 patients (2.1%) were found with homocystinuria from the original 429 intellectually disabled patient cohort. Since, early diagnosis of homocystinuria can help the patients to start a timely treatment and avoid disease pathology. Diet and other pharmacological therapies are available like; pyridoxine in combination with folic acid and vitamin B12; a methionine-restricted, cysteine-supplemented diet, and betaine (41–46). Affected patients can live a normal life by using these treatments, which would indeed be a great relief not only for such patients but also their families.

Aside from classical homocystinuria disease, 2 patients were identified with a suggestive phenotype of MPS, a group of rare IEMs in which the accumulation of glycosaminoglycans (GAGs) occur due to enzyme deficiencies (WES data is in progress to find causative mutations).Two more patients were found with GAMT deficiency, which is a disorder of creatine metabolism amenable to high dose creatine and an arginine restricted diet (47). One patient in the current study showed high concentration of cholesterol, cholestanol, stigmasterol, campesterol and sitosterol confirming sitosterolemia disease. Different therapeutic options are available for the treatment of sitosterolemia like, diets low in plant sterols and shellfish, and use of the sterol absorption inhibitor ezetimibe (48, 49). Early diagnosis is important, especially for those IEMs amenable to treatment as irreversible damage can be prevented, but also for ending the diagnostic odyssey, providing accurate genetic counseling on recurrence risk, identification of family members at risk, and providing an answer and optimizing supportive management. With an early diagnosis, preferably by NBS in the future, such patients can live a healthy life and burden of these kinds of IEMs can be minimized from the society.

## Conclusion

IEMs are rare genetic disorders, and more than 200 of these are potentially treatable if diagnosed early. Diet and pharmacological therapies are available for the treatment of several IEMS. Overall prevalence of IEMs in different population is 2 to 3% and according to the current study, prevalence in the Pakistani cohort was 3.26%, due to high rate of consanguinity. It is worth mentioning that the studied patient cohort did not represent all the geographic locations of Pakistan but only the Faisalabad and Lahore regions of Punjab, Pakistan. Thus, more comprehensive large scale studies covering all areas of Pakistan would be required to know the real prevalence of IEMs. Unfortunately, Pakistan has no NBS program up till now, so this detailed biochemical screening study will help to start an initial setup for NBS program.

In this study, after the biochemical screening, different IEMs have been demonstrated in Pakistani patient cohort, thus diagnoses and treatment options were made available for these rare disorders. It is a practical option with benefits for the patients to start screening of treatable IEMs like aminoacidopathies, fatty acid β-oxidation and creatine metabolic disorders etc. Based on this initial screening data, screening of homocystinuria will be initiated due to its highest frequency (2.1%) among IEMs in the collected 429 intellectually disabled patients from Pakistan. As we have also found 5 patients besides classical homocystinuria, two with MPS, two patients with GAMT deficiency and one with sitosterolemia. In future, we would like to setup a NBS program in Pakistan for the screening of most prevalent IEMs because after the early diagnosis and treatment, affected patients can live long with normal life and have a positive impact on their families.

Following sample collection and initial analysis (HPLC) at NIBGE, Faisalabad, Pakistan, all the advanced biochemical screening was performed in the Laboratory Genetic Metabolic Diseases, Department of Clinical Chemistry, Amsterdam UMC, The Netherlands. This collaborative work will be continued to develop capacity for the IEMs research in Pakistan that will help us to setup a NBS program in Pakistan. After receiving a prompt diagnosis, timely initiation of treatment can help to minimize the deleterious symptoms of these rare IEMs.

## Data Availability

This manuscript contains previously unpublished data. The name of the repository and accession number are not available.

## Ethics Statement

All procedures performed in this study involving human participants were in accordance with the ethical standards of the institutional research committee and with the 1964 Helsinki declaration and its later amendments or comparable ethical standards.

## Consent of Participants

Informed written consent was obtained for all study participants from their parents/head of special education centers.

## Author Contributions

MW and HK collected samples of intellectually disabled patients. MW wrote initial drafts. CvK conceived the idea, revised the manuscript, and approved it. SG and FV have confirmed all the diseases. HA helped in the clinical aspects of this study. FA conducted and supervised this study and revised the manuscript several times and approved it.

### Conflict of Interest Statement

The authors declare that the research was conducted in the absence of any commercial or financial relationships that could be construed as a potential conflict of interest.
